# Interrelationships Among Sensitivity, Precision, Accuracy, Specificity and Predictive Values in Bioassays, Represented as Combined ROC Curves with Integrated Cutoff Distribution Curves and Novel Index Values

**DOI:** 10.3390/diagnostics15040410

**Published:** 2025-02-07

**Authors:** Peter Oehr

**Affiliations:** Faculty of Medicine, University of Bonn, 53113 Bonn, Germany; prof.oehr@gmx.de

**Keywords:** bioassay, diagnosis, UBC^®^ Rapid, ROC curve, PRC–ROC curve, SS–ROC curve, AC–ROC curve, PV–ROC curve, precision, sensitivity, accuracy, specificity, predictive values

## Abstract

**Background/Objectives:** This work introduces accuracy- and precision-ROC curves in addition to SS– and PV–ROC curves and shows a novel means of profiling biomarker characteristics for validation of optimal cutoffs in clinical diagnostics and decision making. **Methods:** This investigation included 91 patients with a confirmed bladder cancer diagnosis and 1152 patients without evidence of cancer. The study performed a quantitative investigation of used-up test cassettes from the visual UBC^®^ Rapid qualitative point-of-care assay, which had already been applied in routine diagnostics. Using a photometric reader, quantitative data could also be obtained from the test line of the used cassettes. The ROC curves were constructed using different thresholds or cutoff levels to determine the TP/TN and FP/FN values for each threshold at concentrations of 5, 10, 30, 50, 90, 110, 250 and 300 µg/L. The resulting TP/TN and FP/FN values were used to calculate the sensitivity/specificity, accuracy, precision and predictive values in order to plot the ROC curves with integrated cutoff value distributions and their index cutoff diagrams. **Results:** A common, optimal cutoff value for all the diagnostic parameters was derived with the aid of an ROC index cutoff diagram. It includes higher specificity and an acceptable number of NPVs. As a result, use of a sensitivity–specificity ROC curve and the Youden index only permits the selection of a maximal threshold value or cutoff point for the biomarker of interest but disregards the clinical status of the patient, whereas the precision, accuracy and predictive values give information related to the disease. **Conclusions:** This work’s novelty compared to the existing methodology includes the first international publication of accuracy- and precision-ROC curves. It enables the investigation of the relationship among the sensitivity, specificity, precision, accuracy and predictive values at varied cutoff levels within a bioassay, presenting these in a single graph consisting of selected receiver operating characteristic (ROC) curves for each parameter, including cutoff distribution curves. This is a transparent method to identify appropriate cutoffs for multiple diagnostic parameters. According to the results, the single-sided use of a sensitivity–specificity ROC curve including the maximal Youden index value as an optimal cutoff or single-point determinations for predictive values cannot provide diagnostic information of the same quality as that given by a multi-parameter diagnostic profile and a multi-parameter cutoff-index-diagram-derived optimal value as presented within this work. The proposed multi-parameter cutoff-index diagram includes novel index cutoff AOX. It is a new different method that allows a quantitative comparison of the results from multi-parameter ROC curves, which cannot be performed with the AUC. However, the methods are different and do not exclude each other.

## 1. Introduction

Laboratory assays for blood-based tumor markers often show considerable overlap between cancer and non-cancer cohorts, requiring a tradeoff between sensitivity and specificity when defining an appropriate cutoff. To directly compare different diagnostic tools, the so-called receiver operating characteristic (ROC) curve has traditionally been used, but it provides limited information about a single biomarker profile and does not include the cutoff distributions across the whole range of possible cutoffs.

Traditional ROC curves were only used for investigations of sensitivity/specificity. They were first developed during World War II to assess the ability of radar operators to differentiate signals (e.g., enemy aircraft) from noise (e.g., a flock of birds), and they were later applied in signal theory, leading to the name “receiver operating characteristic” [1,2,3].

In a traditional ROC curve, the relationship between the sensitivity and specificity is shown by plotting such parameters against one another; when increasing the cutoff concentration value, the sensitivity decreases while the specificity increases. Typically, both values range between 0 and 1, and the sensitivity (true positive (TP) rate) is plotted against 1 minus the specificity (false positive (FP) rate).

The potential of ROC curves for medical diagnostics was first highlighted with regard to radiology by Lusted in 1960 [4]. However, this was only after the publication of the work of Swets and Pickett [5]. During their later extension to other medical fields, these curves were extensively used in radiology to evaluate medical imaging devices [6].

The ROC curve is also known as the relative operating characteristic curve because it is a comparison of two operating characteristics (TP rate and FP rate) at varied cutoff values [7]. The first ROC curves for clinical biomarker diagnosis were introduced in 1981 [8]. They included sensitivity–specificity ROC curves for the diagnostic analysis of in vitro bioassays in patients with tumors (lung, urinary bladder and testicular carcinomas, in comparison to control groups with/without benign diseases). These curves were developed from inverse distribution functions for the improved comparison of biomarker assays through the exclusion of different bioassay concentration parameters, and they were used to avoid the incorrect evaluation of two or more biomarkers when comparing their sensitivities at different specificities, which was common at that time (this article can be obtained via the Internet (Oehr Derigs Altmann ResearchGate)). Initially, the curves were called sensitivity–specificity diagrams, but after finding ROC curves in the literature, the author changed their name to ROC curves as well. At present, ROC curves are also referred to as sensitivity/specificity ROC (SS–ROC) curves because, in recent years, new ROC curves have been introduced.

In 2008, Shiu and Gatsonis were the first to present ROC curves for the joint study of the positive predictive values (PPVs) and negative predictive values (NPVs) of diagnostic tests. Using a mathematical approach, they defined the “Predictive Receiver Operating Characteristic Curve” (PROC) as a curve that consists of all possible pairs of PPVs and NPVs as the threshold for test positivity [9]. Measures of test performance, such as sensitivity, specificity or ROC curves, provide the type of information that is typically needed for technology assessment and health policy purposes. Measures of predictive value provide the type of information that is typically needed for clinical decision making, where clinicians and patients decide whether to use a test or how to assess the implications of a test result [9].

In 2012 and 2020, Special Issues on the application of information theory to epidemiology were published, including further mathematical and the first empirical PV–ROC curves for bioassays [10,11,12,13].

These graphs were plotted without integrated cutoff distribution curves. However, this deficiency could be overcome with the integration of the cutoff value distributions in the sensitivity–specificity/predictive value (SS/PV) ROC and newly established sensitivity–specificity–Youden/predictive value–predictive summary index (SS-J/PV–PSI) ROC curves [14]. While ROC curves without cutoff distribution curves are useful for the direct comparison of different markers and tests, when profiling a biomarker with the inclusion of additional parameters such as precision and accuracy, the concentration distribution curves contribute additional valuable information. This is of particular importance when plotting ROC curves that include both the specificity and NPVs on the *x*-axis of a single graph, because the threshold distribution curves run in opposite directions [14].

This work’s novelty compared to existing methodology presents two new, unpublished ROC curves for the parameters of precision and accuracy. This work is not intended to provide a detailed clinical evaluation of a specific test or biomarker. Rather, the data are used to explain the construction of the novel ROC curves and to demonstrate the different characteristic distributions of all the mentioned ROC curves and their concerted applicability. First, definitions and formulas for the diagnostic parameters are given (Section 2); then, examples of different combinations of ROC curves are presented. This work considers how the sensitivity/specificity, accuracy, precision and predictive values are related to each other within a bioassay and how these relationships change at different cutoff values. The discussion focuses on the importance of using accuracy as a defined diagnostic parameter, instead of examining the “bioassay accuracy” using some combination of single-point PV or sensitivity/specificity values and a traditional ROC curve, including the maximal Youden index as an “optimal cutoff” value. Another section discusses the clinical value of the maximal Youden index as a “diagnostic parameter” compared to the maximal values of the indices for the precision, accuracy and predictive values as an alternative. Finally, a new, diagnostically optimized cutoff value is derived, which takes all indices into account and provides the type of information that is needed for clinical decision making, in contrast to the maximal Youden index alone.

## 2. Methods and Patients

### 2.1. ROC Curves

#### 2.1.1. Definitions

In order to construct the ROC curve, the test’s sensitivity, accuracy, specificity, PPVs and NPVs were assessed according to the following definitions.

Sensitivity: A measure of a diagnostic test’s ability to detect a disease when it is present. It is calculated by dividing the number of TPs (i.e., TP) by the number of patients with the disease, i.e., TPs (i.e., TP) and false negatives (FNs) (i.e., FN): TP/(TP + FN).

Specificity: A measure of a diagnostic test’s ability to identify individuals who do not have the disease. It is calculated by dividing the number of true negatives (TNs) (i.e., TN) by the number of individuals without the disease, i.e., TNs and FPs (i.e., TN + FP): TN/(TN + FP).

Accuracy: A measure of a test’s ability to correctly differentiate between patients and healthy cases. It is calculated using the formula (TP + TN)/(TP + TN + FP + FN).

NPV: The probability of the absence of a disease given a negative test result. It is calculated as the ratio TN/(TN + FN).

PPV and Precision: The PPV, or precision, is a measure of the frequency with which individuals who test positive for the disease actually have the disease. It is calculated by dividing the number of TPs (i.e., TP) by the number of individuals who test positive, i.e., TPs and FPs (i.e., TP + FP), namely, TP/(TP + FP).

The ROC curve is also known as the relative operating characteristic curve because it is a comparison of two operating characteristics (TP rate and FP rate) at varied cutoff values [7]. Within this work, the terms “precision” and “positive predictive value” refer to the same values as a result of their calculation within the same formula. In order to differentiate between them in a graph, i.e., to discern whether a value reflects the specificity or the NPV, two different terms are used. The term “precision” is used in ROC analysis as a function of the specificity, while “PPV” is used in cases as a function of the NPV.

PPV and NPV: The PPV and NPV, respectively, are the proportions of positive and negative results in statistics and diagnostic tests that are TP and TN results, respectively. The PPV and NPV are probabilities that reflect a biomarker’s performance in practice. The PPV considers both the TP and FP biomarker test results and estimates the probability that a disease is present when the biomarker test result is positive. The PPV reflects the likelihood that a person who presents a positive test result has the disease that is being tested in the bioassay.

The ROC curves constructed using different thresholds or cutoff levels were introduced to determine the TP/TN and FP/FN values for each threshold. This was undertaken at concentrations set by the author (5, 10, 30, 50, 90, 110, 250 and 300 µg/L). The resulting TP/TN and FP/FN values were used to calculate the sensitivity/specificity, accuracy, precision and predictive values in order to plot the ROC curves with integrated cutoff value distributions. In these ROC curves, the values for the sensitivity, precision and accuracy were plotted on the *y*-axis, including 1 − specificity on the *x*-axis, whereas, for a predictive ROC curve, the PPVs (=precision) are plotted on the *y*-axis, and those for 1 − NPV are plotted on the *x*-axis instead. Using Microsoft Office Excel 97-2004 software, graphs for the ROC curves were created, including cutoff distribution curves.

#### 2.1.2. SS Index ROC and PV Index ROC Curves

Several indices are available to summarize the information in a ROC graph. In order to determine the optimal cutoffs for the comparison of single or unified evaluations, index ROC curves were developed, using the index suggested by W. J. Youden in 1950 for SS–ROC curves [15] and the predictive summary index (PSI) developed by Linn and Grunau in 2004 for PV–ROC curves [16].

The Youden index is a method applied for the evaluation of optimal cutoff values in SS–ROC curves in order to select the maximum level of sensitivity/specificity [15]. It is defined as Youden index = sensitivity + specificity − 1. This index can take any value between −1 (sensitivity or specificity = 0) and +1 (sensitivity = specificity). The maximum value of the Youden index is 1 (perfect test) and the minimum value is 0, indicating that the test has no diagnostic value. The minimum occurs when sensitivity = 1 − specificity, which is represented by an equal line (diagonal) in the ROC diagram.

The PSI is a tool applied for the evaluation of the optimal cutoff values in PV–ROC curves in order to select the maximum level for the predictive values. The overall gain in certainty can be expressed in the form of the following expression: PSI = PPV + NPV − 1. The maximum value of the PSI is 1 (perfect test), which indicates that the test is always correct and there are no FNs, while for a test that has no predictive value, PSI = 0.

The precision–specificity (PR–SP) index is constructed analogously to the Youden index and PSI. It is introduced by the author for the evaluation of the optimal cutoff values in PR–SP–ROC curves in order to select the maximum level of precision/specificity. It is defined as PR–SP = precision + specificity − 1. This index can take any value between −1 (precision or specificity = 0) and +1 (precision = specificity). The maximum value of the PR–SP index is 1 (perfect test) and the minimum value is 0, indicating that the test has no diagnostic value.

The accuracy–specificity (AC–SP) index is constructed analogously to the Youden index and PSI. It is introduced by the author for the evaluation of the optimal cutoff values in AC–SP–ROC curves in order to select the maximum level of accuracy/specificity. It is defined as PR–SP = accuracy + specificity − 1. This index can take any value between −1 (accuracy or specificity = 0) and +1 (accuracy = specificity). The maximum value of the AC–SP index is 1 (perfect test) and the minimum value is 0, indicating that the test has no diagnostic value.

To ensure systematic terminology (nomenclature) across the literature, including the present and any further developed types of ROC curves and their combinations, and in order to differentiate the Youden index from other novel ROC-curve-related indices, such as those for the predictive values (PV), the term “SS–J index” is used to denote the sensitivity–specificity index introduced by Youden [15], and “PV–PSI” refers to the PSI introduced by Linn and Grunau [16]. Precision–specificity and accuracy–specificity ROC curves have not yet been published in the literature, and they are called PR–SP and AC–SP–ROC curves here.

### 2.2. Patient Data and Clinical Tests

#### 2.2.1. Patients

In order to demonstrate the applicability of the different ROC curves, data from a clinical tumor marker study were used. This study involved a quantitative evaluation using leftover consumables, which are normally discarded and did not include research on humans or animals. Accordingly, ethics committee or institutional review board approval was not required for this study. The aim was to determine whether the quantitative use of the UBC^®^ Rapid test would improve the clinical performance of visual non-quantitative point-of-care (POC) testing in the daily routines of common medical facilities.

A total of 1243 urinary samples from urological facilities in Germany were examined. Prior to sampling, the written consent of the patients was obtained. According to data from routine diagnostics, 91 patients were confirmed to have bladder cancer, and 1152 patients displayed no evidence of bladder cancer, resulting in a prevalence value of 0.073. The exclusion criteria included infections of the urinary tract, invasive treatment and instillations such as BCG, pregnancy and diabetes. Prior to cystoscopic diagnosis (“gold standard”), the diagnostics included quantitative determination via the UBC^®^ Rapid test using the Concile^®^ Ω100 reader, which measured the intensity of the colored line in the POC cassette. For the data evaluation, different band intensity thresholds (cutoff values) for the consideration of a positive test were applied for the determination of FP or FN values and the corresponding sensitivity, specificity and accuracy values.

#### 2.2.2. Tests

The UBC^®^ Rapid biomarker test was used for visual qualitative analysis. The UBC^®^ Rapid test is a commercially available visual POC test that can qualitatively detect antigen fragments of cytokeratin-8 and -18 in urine samples (Arocell, Stockholm, Sweden). The UBC^®^ Rapid test uses qualitative immunochromatographic lateral flow assays, which develop a concentration-dependent color reaction at a cutoff value on a test line at a predefined concentration. A positive reaction was determined according to the subjective interpretation of a human operator.

The UBC^®^ Rapid Concile^®^ Ω100 photometric reader was used for quantitative determination. Photometric readers present an opportunity for the objective, quantitative evaluation of POC assays. This reader is a commercially available measurement device for immediate patient diagnostics and delivers measurement results within 10 to 20 min (Concile GmbH, Freiburg, Germany). The lowest concentration detection level of the UBC^®^ Rapid Concile^®^ Ω100 photometric reader is 0.5 µg/L. For this reason, the resultant distribution curves in this investigation did not include concentration values below the detection limit of the quantitative POC determinations.

Urinstix (leukocyturia, hematuria and nitrite) indicate a possible urinary tract infection. Other examinations are possible, depending on the facility’s routine practices, e.g., urine cytology or ultrasound.

## 3. Results

Table 1 shows the results after the TP/TN and FP/FN values were used to calculate the sensitivity/specificity, accuracy, precision and predictive values at concentrations of 5, 10, 30, 50, 90, 110, 250 and 300 µg/L.

**Figure 1:**
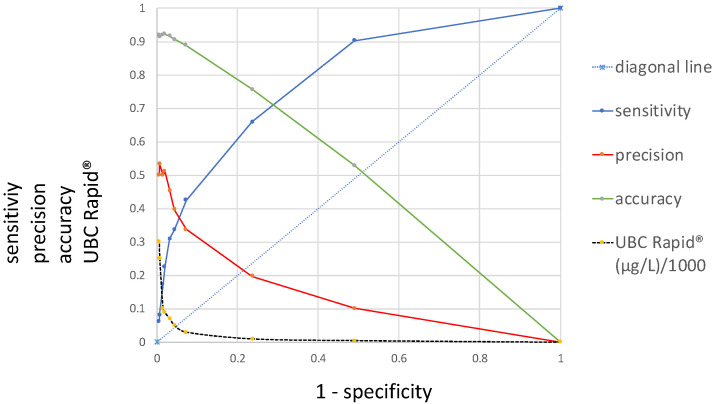
Distributions of precision, accuracy and sensitivity receiver operating characteristic (ROC) curves, including their corresponding UBC cutoff values (*y*-axis) at 1 − specificity (*x*-axis).

Figure 1 presents a novel ROC curve that, in addition to the sensitivity/specificity, displays precision and accuracy as a function of 1 − specificity. Moving from the left side of the graph to the right, from 300 µg/L to 0 µg/L, the sensitivity increases along the *y*-axis from 0 to 1, while the precision and accuracy decrease from 0.5 and 0.9 to 0, diametrically opposed to the sensitivity, along the *x*-axis. In contrast to the curve for the sensitivity, which is located above the diagonal lines and shows an area under the curve (AUC), the precision and accuracy curves intersect with the diagonal. While the curve for precision includes a steep decline at the beginning, the accuracy curve is approximately linear.

**Figure 2:**
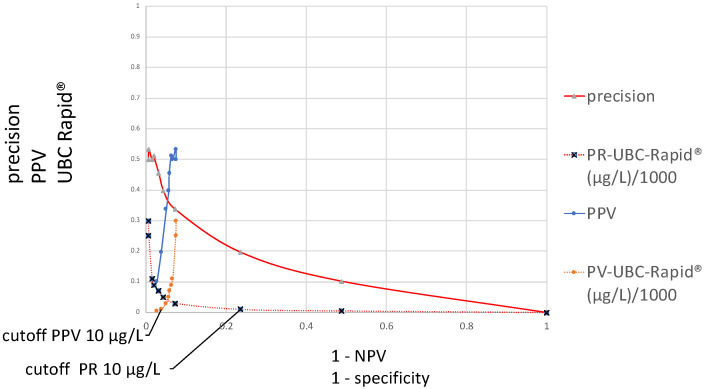
Distributions of precision–specificity and positive predictive values–ROC curves, including their corresponding UBC cutoff values (*y*-axis) at 1 − specificity and 1 − negative predictive values (*x*-axis).

Figure 2 contains two different distribution curves. The values plotted on the *y*-axis are originally the same, both calculated according to precision = PPV, using the formula TP/(TP + FP), and they refer to the same varied cutoff value concentrations. These calculation results are plotted as a function of either the specificity or 1 − NPV. In the first case, the curve is called a precision ROC (PR–ROC) curve; in the second case, it is a predictive value ROC (PV–ROC) curve. While the cutoff distribution curve for the “precision” decreases, the curve runs from the left- to the right-hand side at the bottom of the graph, whereas the values for the “PPV” run in the opposite direction. The distributions of their cutoff values run in opposite directions as well. For these reasons, their respective cutoffs are located at different points, as demonstrated in the figure for a cutoff of 10 µg/L. The distributions and sizes are entirely different. The “precision–specificity” curve extends between 0 and 1 on the *x*-axis and has the maximal “precision” value at approximately 0.5, whereas the “PV–ROC” curve is only located between 0.075 and 0 on the *x*-axis, and the maximal “PPV” value is approximately 0.5 as well.

**Figure 3:**
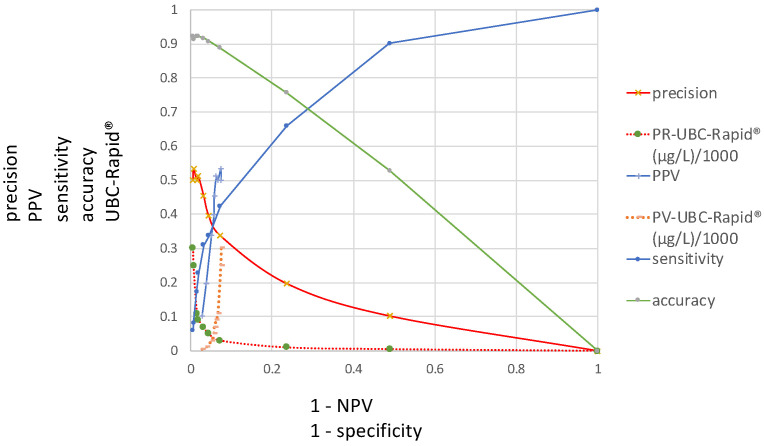
Distributions of sensitivity, precision, accuracy and PPV–ROC curves, including their corresponding UBC cutoff values (*y*-axis) at 1 − specificity and 1 − NPV (*x*-axis).

Figure 3 presents the ROC curves investigated in this study and includes the distributions of the sensitivity, precision, accuracy and PV–ROC curves, including their corresponding UBC cutoff values along the *y*-axis and at 1 − specificity and 1 − NPV on the *x*-axis.

Table 2 contains the values of the specificity, precision, sensitivity, accuracy and predictive indices for the UBC^®^ Rapid test at their corresponding increasing cutoff values from 0.5 µg to 300 µg.

**Figure 4:**
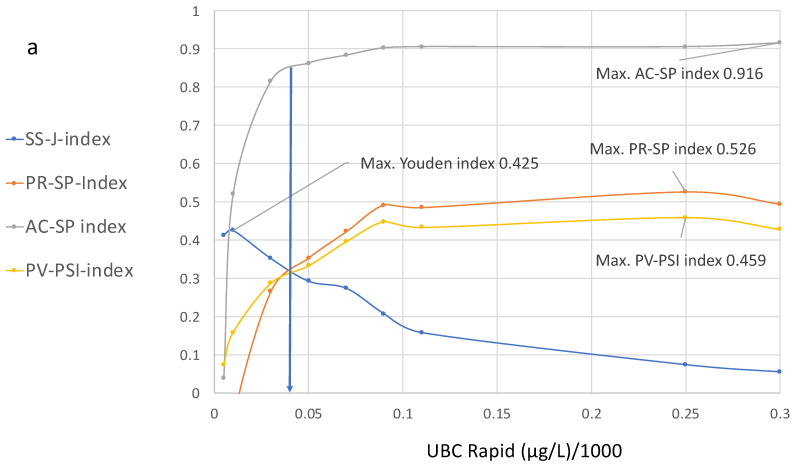

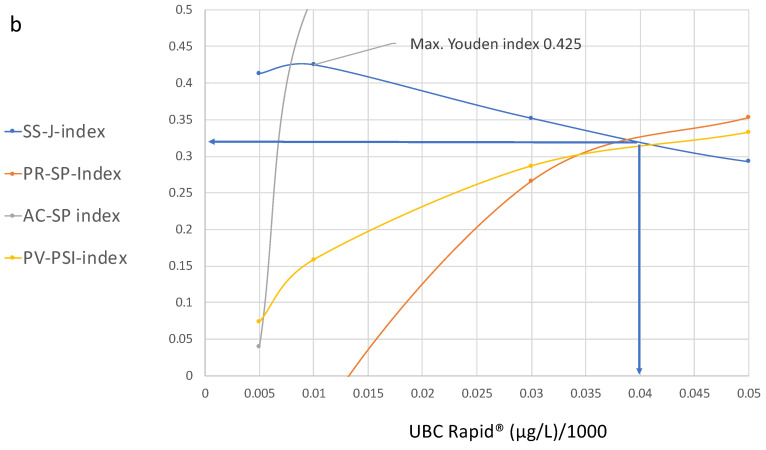
(**a**,**b**) Diagram showing cutoff values plotted on the *x*-axis and the values of the specificity, precision, sensitivity, accuracy and predictive indices for the UBC^®^ Rapid test on the *y*-axis, with their corresponding increasing cutoff values from 0.5 µg to 300 µg. (**b**) is a section of (**a**).

In order to achieve optimized scaling in the corresponding index diagram derived from Table 2, which is shown in Figure 4, a cutoff value of 1/1000 is applied.

Figure 4a,b show the curve distributions derived from Table 2. At increasing cutoff values, the Youden index curve immediately increases to a maximum value of 4.25 at 10 µg/L, followed by a continuous decrease.

In contrast, compared to the maximal value at 10 µg/L, the precision and accuracy values do not display diagnostic utility, while the predictive value–predictive summary index only reaches approximately half of its optimal value. Up to a value of 100 µg/L, the index curves for precision, accuracy and PPV continue to increase; thereafter, the curves become flat and they approach their maximum values at approximately 250 µg/L.

At this cutoff level, the SS–J index is only 0.0558, whereas the PV index is 0.56, the AC–SP index is 0.9 and the PV–PSI is 0.459.

Figure 4b illustrates the cutoff value for the intersection of the SS, PR and PPV curves, which is approximately 40 µg/L. This cutoff corresponds to the following estimated values at 38% sensitivity and 94.2% specificity: SS–J index = 0.32; PR–SP index = 0.31; AC–SP index = 0.84; and PV–PSI = 0.58.

## 4. Discussion and Conclusions

In 1960, the potential utility of SS–ROC curves for medical diagnostics was first observed. During their later extension to other medical fields, these curves became widely used in radiology to evaluate medical imaging devices [4,5,6]. According to Swift, the ROC curve is also known as the relative operating characteristic curve because it presents a comparison of two operating characteristics (TP rate and FP rate) at varied cutoff values [7].

In 2008, Shiu and Gastonis presented a joint study of the PPVs and NPVs of diagnostic tests; this was the first publication in which, using a mathematical approach, the predictive receiver operating characteristic was described in the form of a PROC curve that consisted of all possible pairs of PPVs and NPVs as the threshold for test positivity [9].

Within the present work, two new ROC curves were introduced, concerning precision and accuracy as a function of the specificity, including the curve for sensitivity and the common cutoff distribution curve. Precision reflects the similarity of the measurements to each other, and the accuracy of a test is its ability to correctly differentiate between patients and healthy cases and reflects the overall correctness. Understanding these metrics is crucial for medical diagnostics. Moving from the left side of the graph to the right (Figure 1), with decreasing cutoff concentrations depicted along the *x*-axis, the sensitivity increases while the precision and accuracy decrease. In contrast to the curve for the sensitivity, which is located above the diagonal line and shows an AUC, the precision and accuracy curves intersect with the diagonal and do not have an AUC. Moreover, while the curve for the precision shows a steep decline at the beginning, the accuracy curve is approximately linear.

Figure 2 contains two different ROC curves that display the results of the TP/(TP + FP) calculations, as a function of either 1 − specificity or 1 − NPV. In the case of 1 − specificity, the value distribution is the same as that shown in Figure 1 and it is called the PR–SP–ROC curve. In the case of the function of 1 − NPV, the PV–ROC curve is applied. While the PR–SP–ROC curve is described for the first time in this work, the PV–ROC curve has already been established [9,12,13], and the PV–ROC curve, shown in Figure 2 and Figure 3, was published as part of an SS–PV–ROC curve as well [14]. However, a comparison such as that in Figure 2 and Figure 3, which demonstrates the different properties of ROC curves when the precision (=PPV) is plotted as a function of either the specificity or 1 − NPV, has not been presented before.

The curves for the sensitivity, specificity and predictive values, plotted in Figure 1 and Figure 2, have different values at different cutoff levels. As shown in Table 1, at a cutoff of 10 µg/L, the sensitivity is high (0.66) and the precision is low (0.19); at a cutoff of 50 µg/L, the sensitivity and precision are similar (0.33 and 0.39); and at a cutoff of 90 µg/L, the sensitivity is lower (0.66) than the precision (0.51). This situation becomes even more complex when the cutoff values display the opposite patterns in the graph (Figure 2 and Figure 3). For this reason, the locations of the maximal cutoff values for the sensitivity, precision, accuracy and predictive values will differ in all cases, and four different maximal index values can be calculated. This indicates that the former method of selecting only the maximal Youden index as the optimal diagnostic cutoff value may be insufficient.

Figure 3 includes a summary of all ROC curves described in this study, demonstrating that any of the new ROC curves can be compared at various cutoff levels. This type of figure can be regarded as a diagnostic multi-parameter biomarker profile, which is given in a single graph, and can be used to obtain the necessary information according to the specific diagnostic goals within a clinical study.

Concerning the previous literature on the USB^®^ Rapid bioassay, the authors often calculate the Youden index to determine the “optimal cutoff value”; in addition, they present some single-point determinations for the predictive values with reference to the Youden index.

Styrke et al. [17] calculated an optimal threshold value of ≥8.1 µg/L, resulting in a sensitivity of 70.8%, specificity of 61.4%, a PPV of 71.3% and an NPV of 60.8%. Ritter et al., “using the optimal threshold obtained by receiver operating characteristic analysis (12.3 mg/L)”, stated that the sensitivity, specificity, PPV and NPV of the quantitative UBC^®^ Rapid test were 60.7%, 70.1%, 46.8% and 79.3%, respectively [18]. Pichler et al. [19] reported the best cutoff (highest Youden index; ≥6.7 ng/mL) for the quantitative UBC, which was determined using the ROC curves. For the quantitative UBC^®^ Rapid test, the sensitivity, specificity, PPV and NPV were 64.5%, 81.8%, 71.4% and 76.6%, respectively. According to the data presented in the present work, the maximal Youden index at 10 µg/L leads to 76% specificity.

The comparison of the single-point determinations at selected single cutoff values to determine the diagnostic utility of a test results in insufficient information because, as shown in this study, different curves have different distribution characteristics at various cutoff levels. Accordingly, in single-point determinations for any diagnostic parameter, a fixed specificity or NPV cannot provide relevant information, regardless of whether a maximal Youden cutoff value or any other single-point determination is located within the optimal area of any of the mentioned ROC curves.

The calculation of the maximum Youden index also does not consider whether the result contains a low value for the specificity. Concerning these “optimal cutoff values”, suboptimal specificity is the result. The low specificity of the published “optimal cutoff values” leads to an increased number of FP values, which could result in unnecessary invasive diagnostics in the clinical follow-up of patients suspected to have cancer. Furthermore, the PPV and NPV should not be included based on the optimal value derived from the “receiver operating characteristic analysis for ROC curves describing the sensitivity as a function of the specificity”. Instead, applying the PPV as a function of 1 − NPV is appropriate, e.g., by calculating the PSI [16]. To provide an appropriate, unified threshold for the UBC^®^ Rapid test, threshold estimations from an SS/PV–ROC plot were proposed in 2020 [13] and were later published—these included the maximum unified SS–J/PV–PSI value of 0.32 at the cutoff of 43 µg/L [14].

It is complicated to perform a direct comparison and interpretation of several ROC curves within a single graph, especially when the cutoff distributions run in opposite directions. To solve this problem, the ROC curves shown in Figure 3 were transformed into their corresponding index ROC curves and plotted as an index cutoff diagram, distributed across the complete cutoff range of the quantitative assay. This new tool [14] is useful for the simultaneous comparison and selection of unified or separate optimal cutoff values for all mentioned diagnostic parameters; it can also be used to determine whether a common cutoff level can be applied according to clinical procedures such as diagnosis, follow-up or screening at the optimal specificity and/or NPV. At present, quantitative evaluations are made by calculating the area “over the diagonal”, called the area under the curve (AUC) which is often used as a measure of the test’s performance. Because ROC curves for accuracy and precision or positive predictive values are cutting the diagonal, a comparable AUC cannot be made. The proposed multi-parameter cutoff-index diagram includes novel index cutoff AOX curves. They can be used to make comparative quantitative evaluations by calculating the area over the *x*-axis (AOX) for sensitivity, accuracy, precision and predictive values. It is a new different method that allows a quantitative comparison of results from multi-parameter ROC curves, which cannot be performed with the traditional AUC. However, both methods are different and do not exclude each other. Complete or partial areas across the *x*-axis can be calculated for summarized quantitative effectivity evaluations, with respect to single and/or unified indices and single, separate or several unified cutoff thresholds. This offers an alternative to the AUC, which can only be derived from an SS–ROC curve.

It can be seen that the maximal values for the Youden index do not overlap with the maximal values of the other indices. However, according to the curve distributions, a common optimal cutoff value for all diagnostic parameters could be derived that includes higher specificity and an acceptable number of NPVs. This value was found at the intersection of the SS, PR and PPV curves at approximately 40 µg/L and corresponds to the following estimated values at 38% sensitivity and 94.2% specificity: SS–J index, 0.32; PR–SP index, 0.31; AC–SP index, 0.84; and PV–PSI, 0.58 (Figure 4b).

One of the goals of this study was to introduce new ROC curves for precision and accuracy in order to evaluate their relationships with the previously published SS– and PV–ROC curves, including various cutoff distribution values. This was intended to reveal whether they could share a common optimal cutoff value and whether a single maximum Youden index is still the optimal choice.

Further goals were to demonstrate that the present practice involving the evaluation and characterization of quantitative diagnostic assays by including a ROC curve and single-point determinations derived from a clinical study cannot provide diagnostic information of the same quality as that offered by a multi-parameter diagnostic profile or a cutoff index diagram, as well as to propose a transparent method to identify appropriate cutoffs for multiple diagnostic parameters.

The final goal was to demonstrate that the use of individualized definitions for the characterization of diagnostic parameters leads to confusion in the scientific literature and should be avoided in the future. With respect to the data presented in this work, the use of the term “accuracy” was selected for the UBC^®^ Rapid test for bladder cancer.

In statistics, the definition of “accuracy” is governed by the International Organization for Standardization (ISO) and is as follows: “Accuracy is the proximity of measurement results to the true value” [20]. In the present work, “accuracy” refers to “diagnostic accuracy” and is expressed as the proportion of correctly classified subjects among all subjects. It is calculated using the formula (TP + TN)/(TP +TN + FP + FN). However, many scientific or medical publications, when confronted with the task of defining “accuracy”, refer to the common household definition (i.e., it is “the quality of being correct or true to some objective standard”). In the more recent literature, the ISO is used to define “accuracy”, particularly in publications concerning bioassays related to urinary bladder cancer. Here, an example is given for the common use of this term, from the publication “Evaluation of the diagnostic accuracy of UBC^®^ Rapid in bladder cancer: a Swedish multicentre study” [17]. According to the authors, the aims of their study were to investigate the diagnostic accuracy of the UBC^®^ Rapid test in patients with primary bladder cancer, patients with a history of bladder cancer, those with a benign urological disease and healthy controls, based on the optimal cutoff value for the study population. They also sought to compare the test results in high- and low-risk urothelial tumors. The accuracy was described in terms of sensitivity, specificity and predictive values. The diagnostic accuracy of the UBC^®^ Rapid test in all cases was provided in tables based on cutoffs for the sensitivity, specificity, PPV and NPV. One of the published figures provided results for the PPV of the UBC^®^ Rapid at four different threshold concentrations. The “primary cutoff value” (also called the “optimal cutoff”) used for the calculation of the “diagnostic accuracy” and the predictive values was “based on an optimal cutoff (receiver operator characteristics curve analysis)”. However, according to ISO 5725-1 [20], the general term “accuracy” is used to describe the closeness of a measurement to the true value, whereas optimal cutoffs such as the Youden index, derived from SS–ROC curves, measure the effectiveness of a diagnostic marker and permit the selection of an optimal sensitivity/specificity threshold value or cutoff point for the biomarker of interest. They cannot be used as a measure of accuracy, and this is also the case for predictive values.

Although the new methods described in this manuscript represent progress in the evaluation of bioassays, prevalence as a limiting factor should be mentioned. The PV–ROC curves from different studies including different prevalences cannot be directly compared with respect to the precision, accuracy and predictive values, because their curve distributions vary at different prevalences [9,12]. According to preliminary, unpublished data collected by the author of this manuscript, this is the case for the PRC– and AC–ROC curves as well. The prevalence value of 0.073 mentioned in this study reflects the daily routines of urological facilities and can already be regarded as valuable information for the practicing urologist.

PROC curves for different prevalence values may allow a preview of the likely extent of differences between the curves for subgroups. For example, the prevalence of bladder cancer is known to differ between subgroups of males and females [13]. In such a situation, an array of more than one prevalence value is deemed necessary. PROC curves for different prevalence values may allow a preview of the likely extent of differences between the curves for each of the subgroups [12]. Because of the dependence of PROC curves on prevalence, Hughes et al. displayed an array of PROC predictive values. The PROC curves for different prevalence values may allow a preview of the likely extent of differences [12]. For this reason, the construction of arrays for precision and accuracy ROC curves is an important task for extending the applications of the new methods described in this work.

The mechanisms by which the curves may change their distributions remain to be investigated. Nevertheless, the new methods presented in this manuscript are applicable for the comparison of different biomarkers within the same study groups, and they are also applicable in other fields of science, e.g., plant epidemiology, machine learning algorithms and neural networks, AI and the economy.

## Figures and Tables

**Table 1:** Values for specificity, precision, sensitivity, accuracy and predictive values for the UBC^®^ Rapid test at their corresponding increasing cutoff values from 0.5 µg to 300 µg. In order to achieve optimized scaling in the receiver operating characteristic (ROC) plot, the cutoff/1000 value was used.

UBC^®^ Rapid µg/L	Sensitivity	Specificity	Precision = PPV	Accuracy	NPV
5	0.90	0.51	0.10	0.52	0.97
10	0.66	0.76	0.19	0.75	0.96
30	0.42	0.92	0.33	0.88	0.94
50	0.33	0.95	0.39	0.90	0.94
70	0.30	0.96	0.45	0.91	0.94
90	0.22	0.98	0.51	0.92	0.94
110	0.17	0.98	0.5	0.92	0.93
250	0.08	0.99	0.53	0.91	0.92
300	0.06	0.99	0.5	0.92	0.92

**Table 2:** Values of specificity, precision, sensitivity, accuracy and predictive indices for the UBC^®^ Rapid test at their corresponding increasing cutoff values from 0.5 µg to 300 µg.

UBC^®^ Rapid µg/L	Sensitivity	Specificity	SS-J Index	PR-SP Index	AC-SP Index	PV-PSI Index
5	0.90	0.51	0.41	−0.38	0.04	0.07
10	0.66	0.76	0.42	−0.07	0.52	0.15
30	0.42	0.92	0.35	0.26	0.81	0.28
50	0.33	0.95	0.29	0.35	0.86	0.33
70	0.30	0.96	0.27	0.42	0.88	0.39
90	0.22	0.98	0.20	0.49	0.90	0.44
110	0.17	0.98	0.15	0.48	0.90	0.43
250	0.081	0.99	0.07	0.52	0.90	0.45
300	0.061	0.99	0.05	0.49	0.91	0.42

## Data Availability

The documents containing the clinical data are held by the author and are stored in compliance with data protection regulations.
